# Emerging hypervirulent *Klebsiella pneumoniae*: Myth, reality, and clinical relevance

**DOI:** 10.1016/j.imj.2026.100272

**Published:** 2026-06-30

**Authors:** Sanika Mahesh Kulkarni, Jobin John Jacob, Abi Manesh, Balaji Veeraraghavan

**Affiliations:** a Department of Clinical Microbiology, Christian Medical College and Hospital, Vellore, 632004, India; b The Tamil Nadu Dr. M.G.R. Medical University, Guindy, Chennai, 600032, India; c Department of Infectious Diseases, Christian Medical College and Hospital, Vellore, 632004, India

**Keywords:** Hypervirulent *Klebsiella pneumoniae*, Convergent clone, Lineage-specific virulence, Antimicrobial resistance, Pathotype

## Abstract

The global emergence of hypervirulent *Klebsiella pneumoniae* (hvKp) has raised concern because of its association with invasive infections and reports of increased mortality. However, recent systematic reviews and meta-analyses reveal a critical paradox: genotypically defined convergent clones and strains carrying fewer virulence-associated markers exhibit mortality rates comparable to, rather than exceeding, those of multidrug-resistant (MDR) *K. pneumoniae*. In contrast, traditional phenotypic identification preferentially enriches for the classical hypervirulent lineages that remain uniquely associated with metastatic complications. Genome-wide association studies (GWAS) and phenotypic analyses further confirm capsular polysaccharide as a pivotal, plasmid-independent virulence driver in carbapenem-resistant hvKp cohorts, with only 37.3% (22/59) demonstrating true hypervirulence despite universal plasmid marker presence, reinforcing lineage-specific risk over gene carriage alone. The definitional crisis, wherein virulence gene carriage alone fails to predict clinical outcomes, highlights the importance of lineage-specific risk. This is best exemplified by K1/K2 sequence type (ST)23, including clonal group (CG)23-like shadow lineages such as ST65, ST86, ST412 and ST268, which are consistently associated with metastatic disease and adverse outcomes in otherwise healthy hosts. Synthesizing clinical, genomic, and epidemiological evidence, this perspective proposes an updated *K. pneumoniae* pathotype framework that integrates clonality, antimicrobial resistance (AMR), and epidemiological context; advocates tiered laboratory diagnostics; and cautions against indiscriminate hvKp labelling to safeguard antimicrobial stewardship and informed clinical decision-making.

## Graphical abstract

Created using BioRender.com under a licensed account held by the corresponding author. Publication license documentation is provided.

**Figure:**
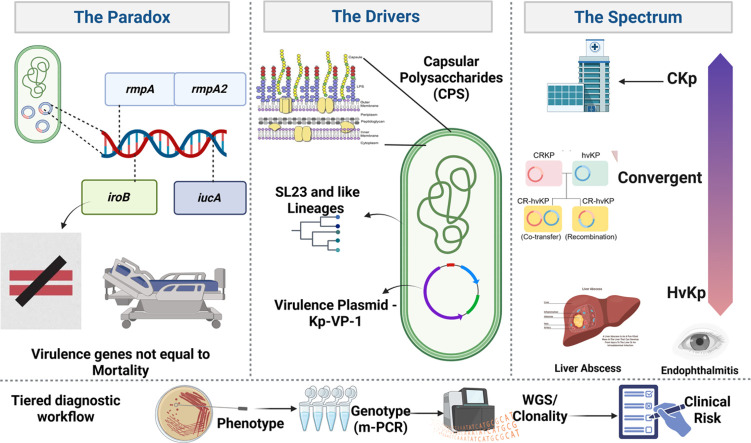

## 1 Introduction

The global emergence of hypervirulent *Klebsiella pneumoniae* (hvKp) has generated substantial concern due to its association with invasive infections, metastatic complications, and reports of increased mortality.^1^^,^^2^ However, accumulating evidence from recent systematic reviews and meta-analyses highlights a fundamental disconnect between how hypervirulence is defined and how clinical risk, particularly mortality, is interpreted.^1^^,^^2^ Across studies, hvKp has been variably identified using phenotypic markers such as hypermucoviscosity (string test) or through genotypic detection of virulence-associated genes, resulting in substantial heterogeneity in reported outcomes.

The contrasting phenotypic and genotypic definitions of hvKp, together with their respective strengths and limitations, are summarized in Table 1. The absence of a harmonised definition has obscured distinctions between historically hypervirulent community-associated lineages and newly emerging virulence-associated hospital strains, thereby fueling uncertainty regarding the true clinical relevance of hvKp.^3^^,^^4^

**Table 1:** Definitions of hypervirulent *Klebsiella pneumoniae*: Phenotypic vs. genotypic approaches, synthesized from published diagnostic and classification literature.

Definition approach	Criteria used	Strengths	Key limitations	Clinical implication
Phenotypic (String test)^4^, ^5^, ^6^	Hypermucoviscosity (> 5 mm)	Enriches for classical K1/K2 ST23 hvKp	Low sensitivity; false positives in MDR cKp	Identifies a small but high-risk subset
Genotypic (PCR-based)^6^, ^7^, ^8^	Detection of *iucA, rmpA, rmpA2, iroB, peg-344*	High sensitivity; scalable for surveillance	Gene presence ≠ expression or virulence	Overestimates clinical risk
CPS quantification^10^	CPS production or *rcsA* expression	Independent biomarker; plasmid-free	Requires specialized assays	Discriminates true hv-CRKp
Combined approach^11^^,^^12^	PCR + string test + capsular typing + whole-genome sequencing	Best risk stratification	Resource intensive	Recommended for reference labs

*Abbreviations*: cKp, classical *K. pneumoniae*; CPS, capsular polysaccharide; hv-CRKp, hypervirulent carbapenem-resistant *Klebsiella pneumoniae*; hvKp, hypervirulent *Klebsiella pneumoniae*;MDR, multidrug-resistant; PCR, polymerase chain reaction.

Over the past decade, hvKp epidemiology has been further complicated by a bidirectional convergence between virulence and antimicrobial resistance (AMR).^3^^,^^5^ Contemporary operational definitions frequently classify hvKp as isolates carrying ≥ 4 virulence determinants, most commonly *iuc, iro, rmpA, rmpA2*, and *peg-344.*^4^^,^^6^, ^7^, ^8^ Within this framework, “convergent hvKp” refers to classical hypervirulent lineages that have acquired extended-spectrum β-lactamase (ESBL) or carbapenem-resistance determinants, whereas “convergent classical *Klebsiella pneumoniae* (convergent-cKp)” describes nosocomial multidrug-resistant or carbapenem-resistant clones that have acquired partial virulence plasmids.^3^^,^^9^

As illustrated in Table 1, phenotypic approaches preferentially enrich for historically virulent K1/K2 lineages, whereas genotypic screening captures a broader and more heterogeneous population. Importantly, virulence gene detection alone does not reliably predict invasive disease or mortality.^1^^,^^2^^,^^11^ This definitional ambiguity gives rise to two central paradoxes shaping contemporary understanding of hvKp clinical impact.

**Paradox 1: Emerging hvKp and mortality risk.**

Recent meta-analyses have rigorously evaluated the extent to which genotypic markers of hvKp independently drive increased mortality. When hvKp is defined genotypically based solely on the detection of virulence genes such as *iuc, iro, rmpA*, and *peg-344* pooled analyses consistently demonstrate mortality rates comparable to, rather than exceeding, those of carbapenem-resistant *Klebsiella pneumoniae* (CRKp).^1^^,^^2^ These findings indicate that virulence gene carriage alone is insufficient to explain adverse outcomes, a conclusion supported by complementary evidence from experimental models highlighting variability in functional virulence expression. A structured summary of the different levels of evidence and their key interpretations is provided in Table 2.

**Table 2:** A structured summary of the different evidence levels and their key interpretations.

Study type	Example studies	Key finding	Interpretation
Meta-analyses	Chen et al.,^1^ Nagendra et al.^2^	No consistent independent increase in mortality	Virulence gene carriage alone is insufficient
Observational clinical studies / cohort studies	Lee et al.,^5^	Phenotypically defined hvKp is associated with severe disease	Likely reflects enrichment of high-risk lineages
Experimental / animal models	Xu et al.,^10^ Stanton and Wyres, Yang et al.^11^^,^^12^	Only a subset exhibits true hypervirulence despite gene carriage	Functional validation required

*Abbreviation*: hvKp, hypervirulent *Klebsiella pneumoniae*.

Recent profiling of CRKp isolates (with *iuc/iro/rmpA/rmpA2/peg-344)* identified only 37.3% (22/59) as truly hypervirulent mouse median lethal dose (LD50): ≥50% mortality at 1 × 10⁷ colony-forming units (CFU). Genome-wide association studies revealed capsular polysaccharide (CPS) production and expression of the capsule-regulatory gene *rcsA* as predictors, with an area under the curve (AUC) of 0.978, underscoring chromosomal CPS dominance over plasmid-borne virulence determinants.^10^

In contrast, studies employing phenotypic definitions, particularly the string test for hypermucoviscosity combined with all virulence markers, report significantly higher mortality rates.^1^^,^^2^^,^^5^ This discrepancy reflects enrichment for specific high-risk or classical virulent lineages rather than a universal hypervirulence effect. A summary of pooled mortality estimates and interpretations is provided in Table 3. Importantly, the 37.3% figure, while derived from a single study, is consistent with emerging experimental data. Recent studies demonstrate that isolates carrying both resistance and virulence determinants, including near-complete virulence plasmids, do not consistently exhibit hypervirulent phenotypes in experimental models, and that virulence gene presence does not equate to functional expression, highlighting the role of regulatory, genetic, and host context while underscoring the need for cautious interpretation of hypervirulence in clinical practice.^11^^,^^12^ This distinction between gene detection and phenotypic virulence is further compounded by transcriptional regulation, gene–gene interactions, and host immune context. Moreover, current phenotypic assays, including the string test, have well-recognised limitations in the context of carbapenem-resistant hvKp: string test positivity is neither sensitive nor specific for true hypervirulence across lineages, and many virulence plasmid–carrying strains may lack hypermucoviscosity despite harbouring these determinants, underscoring the need for refined molecular and phenotypic diagnostics beyond conventional screening.

**Table 3:** Mortality outcomes of hypervirulent vs. classical *Klebsiella pneumoniae*.

Study	Comparison	Pooled or for mortality (95% CI)	Statistical significance	Key interpretation
Chen et al.^1^	CR-hvKp vs. CRKp	2.05 (0.89–4.75)	Not significant	Mortality not intrinsically linked to hvKp
	Genotypic hvKp vs. cKp	1.05 (0.35–3.16)	Not significant	Virulence genes alone do not predict death
	Phenotypic hvKp vs. cKp	4.16 (1.16–14.92)	Significant	Reflects enrichment of ST23 K1/K2
Nagendra et al.^2^	hvKp vs. cKp (all isolates)	1.20 (0.93–1.55)	Not significant	hvKp causes invasive disease, not excess mortality
Xu et al.^10^	CPS-high hv-CRKp vs. CRKp	Or not reported; sepsis 72.7% vs. 43.2% (*p* = 0.028)	Significant for sepsis	CPS drives severity independent of plasmids

*Abbreviations*: CI, Confidence interval; CPS, capsular polysaccharide; CR-hvKp, carbapenem-resistant hypervirulent *Klebsiella pneumoniae*; CRKp, carbapenem-resistant *Klebsiella pneumoniae*; hv-CRKp, hypervirulent carbapenem-resistant *Klebsiella pneumoniae*; hvKp, hypervirulent *Klebsiella pneumoniae*.

**Paradox 2: The K1/K2 sequence type (ST)23/clonal group (CG)23exception—true hypervirulence**.

The second paradox is exemplified by the K1/K2 ST23/CG23 lineage, which represents the archetype of true hypervirulence. Unlike recently emerging convergent hvKp strains, ST23 and closely related CG23 variants consistently demonstrate elevated mortality and a pronounced propensity for metastatic complications.^4^^,^^14^^,^^15^ This clinical severity is driven by conserved virulence plasmids, enhanced serum resistance, and a strong predilection for community-acquired invasive disease, often occurring in otherwise healthy individuals.^4^^,^^14^

Notably, even within ST11-K Locus(KL)64/ K Locus 47 convergent strains (China-endemic CRKp lineages), CPS production levels determined virulence divergence independent of plasmid content. Strains with higher CPS exhibited greater phagocytosis resistance, higher sepsis incidence (72.7% vs. 43.2%, *p* = 0.028) reinforcing that CPS expression drives clinical severity.^10^

Clinically, these infections characteristically present as pyogenic liver abscesses with frequent progression to endophthalmitis or central nervous system involvement. Phenotypically, ST23 isolates display classical hypermucoviscosity on string testing and produce high-affinity siderophores, including aerobactin and salmochelin.^4^^,^^14^ Although ST23 remains the global reference lineage for hvKp, genomic surveillance has identified an expanding cluster of ST23-like “shadow lineages” such as ST412, ST268, ST25, ST65, ST86, ST218, and ST29 that retain a similar virulence architecture.^15^ These observations suggest that true hypervirulence is strongly influenced by clonal background and lineage context, rather than being defined solely by virulence gene counts.^4^^,^^15^

## 2 Updated pathotype framework

To reconcile microbiological diversity with clinical relevance, an updated classification framework for *Klebsiella pneumoniae* pathotypes is proposed as a conceptual framework intended to guide clinical risk stratification and future empirical validation, integrating clonal background, virulence gene carriage, CPS production, AMR profile, epidemiological context, and dominant drivers of mortality.^1^, ^2^, ^3^ CPS is highlighted as an important contributor; however, virulence is multifactorial and context-dependent, arising from interactions among CPS, siderophore systems, regulatory networks such as *rmpA/A2*, and host immune status.^11^^,^^12^ This framework should be interpreted within a broader functional validation paradigm: Phenotype-informed classification rather than gene-count thresholds alone should anchor future cohort studies, genomic-clinical correlation analyses, and experimental validation to prospectively assess the framework’s predictive performance for clinical outcomes. The proposed framework and its clinical correlates are summarized in Table 4.

**Table 4:** Proposed updated pathotype framework for *Klebsiella pneumoniae* with clinical correlates, derived by published clinical, genomic, diagnostic, and functional evidence.

Pathotype	Typical clone (ST)	Virulence genes	Resistance genes	CPS production	String test	Usual setting	Mortality driver
cKp MDR^1^^,^^4^^,^^6^	ST11, ST15, ST147, ST231, etc.	Absent	Extensive	Low	Negative	ICU/hospital	Resistance + comorbidities
hvKp^4^^,^^6^^,^^11^	ST23, ST65, ST86	Present	Absent	High	Positive	Community	Intrinsic virulence + CPS
Convergent hvKp (acquired MDR)^2^^,^^3^^,^^7^^,^^9^	ST23 + resistance	Present	Present	Variable-High	Mixed	Both	Virulence + resistance
Convergent cKp (acquired virulence)^1^^,^^10^^,^^12^^,^^13^	ST11/ST15 + virulence genes	Present (often truncated)	Extensive	Variable-Low	Often negative	Nosocomial	Host factors, ICU care

*Notes:* Virulence genes include *iuc* (aerobactin), *iro* (salmochelin), and *rmpA/A2* (mucoid regulators). Resistance genes include Carbapenemases (*bla*_KPC_, *bla*_NDM_, *bla*_OXA-48-like_) and ESBLs (*bla*_CTX-M_). Current operational definitions often use ≥4 virulence markers to classify hvKp; however, this proposed framework emphasizes that gene-count thresholds should be interpreted alongside clonal background, resistance profile, CPS production, and phenotypic validation.

*Abbreviations*: cKp, classical *Klebsiella pneumoniae*; CPS, capsular polysaccharide; ESBL, extended-spectrum β-lactamase; hvKp, hypervirulent *Klebsiella pneumoniae*; MDR, multidrug-resistant; ST, sequence type.

## 3 Implications for clinical microbiology and practice

For diagnostic laboratories, indiscriminate reporting of hvKp based solely on string test positivity or polymerase chain reaction (PCR) detection of virulence genes without capsular typing or clonal context provided by whole-genome sequencing risks overestimating clinical severity.^6^^,^^13^ Integration of CPS quantification or *rcsA* expression assays may improve discrimination of truly hypervirulent CRKp strains from genotypically positive but phenotypically attenuated convergent strains.^10^ A tiered diagnostic strategy incorporating molecular screening, capsular typing, selective phenotypic confirmation, and consideration of CPS biomarkers is therefore recommended.

A laboratory-to-bedside risk stratification algorithm translating microbiological findings into actionable clinical guidance is provided in Table 5. This approach prioritizes vigilance for classical K1/K2 hvKp while avoiding unnecessary escalation for convergent nosocomial strains.^1^^,^^2^^,^^13^

**Table 5:** Practical laboratory and clinical risk stratification algorithm for actionable hvKp clinical guidance, summarized from published diagnostic and clinical evidence.

Laboratory finding	Interpretation	Recommended clinical action
String test negative, PCR-negative for virulence genes^4^^,^^6^	cKp	Standard management
String test positive, PCR-positive, K1/K2 capsule, susceptible, ST23/ST65/ST83 or ST23-like^4^^,^^5^^,^^14^	hvKp	Screen for metastatic infection
String test positive or negative, PCR-positive, non-K1/K2 capsule^1^^,^^6^^,^^15^	Convergent hvKp	Treat as severe healthcare-associated infection (HAI)
String test positive or negative, PCR-positive, K1/K2 positive, AMR positive^2^^,^^7^^,^^8^	Convergent hvKp (ST23/ST65/ST86-like lineages)	Heightened vigilance, stewardship-guided therapy
High CPS production or *rcsA* upregulation in CRKp^10^, ^11^, ^12^	Likely true hv-CRKp	Vigilance for invasive disease; consider as high-risk

*Abbreviations*: AMR, antimicrobial resistance; cKp, classical *Klebsiella pneumonaie*; CPS, capsular polysaccharide; CRKp, carbapenem-resistant *Klebsiella pneumoniae*; hv-CRKp, hypervirulent carbapenem-resistant *Klebsiella pneumoniae*; hvKp, hypervirulent *Klebsiella pneumoniae*; PCR, polymerase chain reaction.

## 4 Conclusions

Data summarized in Tables 1,Table 3, Table 4, Table 5 reinforce a central conclusion: Not all hvKp carries equal clinical risk. Mortality is driven by lineage-specific virulence, chromosomally encoded capsular polysaccharide production, AMR, and host context rather than by the presence of virulence genes alone. CPS is a critical independent determinant, but virulence remains multifactorial, shaped by siderophore systems, regulatory elements, gene expression levels, and host immune environment.^11^^,^^12^ Aligned with the evolving consensus that hvKp requires phenotype-informed classification rather than gene-count thresholds,^11^^,^^12^ this perspective cautions against indiscriminate hvKp labelling based on genotypic markers alone. The pivotal role of CPS as an independent, plasmid-free virulence determinant highlights the urgent need for phenotypic validation and biomarker-driven diagnostics in carbapenem-resistant strains. Refining laboratory definitions and reporting practices is therefore essential to improve clinical decision-making and antimicrobial stewardship.

## CRediT authorship contribution statement

**Sanika Mahesh Kulkarni:** Writing – original draft, Visualization, Conceptualization. **Jobin John Jacob:** Writing – review & editing, Investigation, Conceptualization. **Abi Manesh:** Writing – review & editing, Resources, Investigation. **Balaji Veeraraghavan:** Writing – review & editing, Supervision, Project administration, Conceptualization.

## Informed consent

Not applicable.

## Organ donation

Not applicable.

## Ethical statement

Ethics approval was waived for this work because no patients’ data were reported.

## Animal treatment

Not applicable.

## Generative AI

No generative AI tools were used to generate scientific content, data, or analyses for this manuscript. Language editing and drafting support were reviewed and verified by the authors, who take full responsibility for the final content.

## Funding

This work was supported by the Internal Fluid Research Grant of Christian Medical College and Hospital , Vellore. ID: 15248, dated: 22.03.2023.

## Declaration of competing interest

The authors declare that there are no affiliations with or involvement in any organization or entity with any financial interest or non-financial interest in the subject matter or materials discussed in this manuscript.

## Data Availability

Data sharing is not applicable to this article as no new datasets were generated or analyzed.
